# Rethinking Performance Measurement of Primary Care in China: Comment on "Quality and Performance Measurement in Primary Diabetes Care: A Qualitative Study in Urban China"

**DOI:** 10.34172/ijhpm.2023.7825

**Published:** 2023-08-15

**Authors:** Jin Xu

**Affiliations:** China Center for Health Development Studies, Peking University, Beijing China

**Keywords:** Performance Measurement, Primary Care, Non-communicable Diseases, China

## Abstract

Increased political commitment and financial input to primary care have led to a growing role of performance measurement. Rasooly et al studied the implementation of performance measurement for primary care for people with diabetes in China. This is an important topic that has received little attention from previous literature. In light of the findings from the article, this paper argues for rethinking the current use of performance measurement. It also suggests potential ways to improve primary care performance measurement, in order to avoid some of the pitfalls of top-down performance measurement and to create an enabling environment for primary care strengthening.

 Well-functioning primary care is widely considered the corner stone of a high-performing health system. Along with increased political commitment and financial input to primary care, various tools to measure the performance of primary care has been developed by national and international agencies to facilitate establishment of accountability.^1-5^ Some of these tools were also applied in payment for primary care facilities, ie, “pay-for-performance.” However, the results of performance measurement (particularly as a basis for paying healthcare providers) were mixed.^6^ In 2009, China launched a comprehensive health system reform that positioned primary care strengthening among its top priorities. A range of performance indicators for primary care have been used, with many tied to salary for primary care providers. In light of a recent paper^7^ published in the *International Journal of Health Policy and Management* and the findings from others, we discuss the way forward for using primary care measurement in China.

## Growing Role of Performance Measurement in China

 The comprehensive health system reform in China led to rapidly growing public investment in primary care in the country. Through the establishment of Basic Public Health Service Scheme,^8^ an increasing amount of spending went into a range of public health services provided by primary care facilities including diabetes management, health management for elderly, maternal and child health, health records and so on. The Urban-Rural Basic Social Health Insurance Scheme also increased reimbursement for primary care services. Reimbursement rates for outpatient visits by patients with diabetes and a range of other non-communicable diseases (NCDs) at primary care facilities were substantially increased (from about 50% to about 80%). This increased public expenditure led to the valid question about “value for money.” Besides, an ambitious scheme of family doctor contract services was also introduced to improve the coordination and continuity of care. In response to the expanding spending and reform among others, the role of performance measurement has also substantially grown. Due to the importance of hypertension and diabetes as key mitigable risk factors for cardiovascular disease (CVDs) — the top cause of mortality and morbidity in China, management for hypertension and diabetes feature prominently in these performance measures.

 Performance measurement takes place at multiple levels. At its maximum, there may be as many as 7 levels of monitoring and evaluation — namely, from individual health worker, teams of family medicine, primary care facilities, to local county/district, municipal, provincial, and national levels. Indeed, a typical scene in a rural township health center (or an urban community health center) is a big board glued to the wall of the director’s office. On the board is a large table including a range of performance indicators that cover both outpatient services and basic public health service functions including management of hypertension and diabetes patients among others. The table is well aligned if not exactly the same with the national guideline for performance evaluation.

## Strengths and Limitations of a Top-Down Approach

 Performance on key indicators is evaluated and ranked at each level of measurement, giving rise to both hierarchical and peer pressure to excel or to keep up with others. Moreover, pay-for-performance is also established for such services. In other words, both financial incentive and peer-pressure contributed to a powerful structure that turned funding and resources into activities of NCDs management. This top-down structure channels the centrally defined targets all the way to the level of primary care in each township and village, expanding service coverage rapidly across the country. Significant progress has been reported in improvement in care for hypertension and diabetes,^9,10^ as well as reduced patient expenditure.^11^ However, such progress was confronted by the continuous growth in mortality attributable to CVDs, as well as the widening gaps between urban and rural CVDs mortality rates,^12^ suggesting that there is still much room for improvement in the quality of NCDs management.

 The study by Rasooly et al^7^ shed lights on the implementation story behind the numbers. The hierarchical structure of performance measurement accompanied with frequent monitoring and evaluation has contributed to the implementation of the reform. The clear targets have also facilitated clarification of the work of frontline health workers and even help create a sense of collaboration both within primary care facilities and between primary care doctors and hospital specialists. In short, the use of stringent performance measure not only helps make frontline providers answerable to the multilevel hierarchy but also provides room for local collaboration.

 On the other hand, the study revealed limitations in the current use of performance measurement. First, there has been little room for bottom-up feedback from frontline practitioners and middle-level managers during planning of the performance targets. Second, as the gaps in patients’ trust of primary care doctors persists, leading to continued bypassing of primary care by patients who often opt to seek care directly from hospitals. Third, the rigid performance indicators also have done little to reflect the patients’ actual needs for health and wellbeing and may lead to replacement of internal motivation for doctors to address the need of patients. Fourth, important gaps in performance measurement for primary care services, including lack of use of guideline recommended effective measures due to absence of public finance to cover fees for the tests. Fifth, the authors also identified fraudulent reporting despite mechanisms for verification. This lack of effective verification coupled with the incentive to excel in performance is likely not restricted to the local area. Worryingly, fraudulent reporting may lead to systemic over-reporting of performance, pushing further for unrealistic and inflated targets, cherry-picking of patients easier to manage, and concealing of real service gaps.

## Way Forward

 Newton-Lewis et al^13^ have pointed out that the complex and dynamic nature of health systems makes outcome of health services difficult to control. The predominantly directory use of performance measurement widely seen in low- and middle-income countries should be shifted towards more enabling approaches. As Newton-Lewis et al^13^ highlighted, system-level environment and organizational culture play important roles in determining the appropriate balance between directive and enabling approaches. Building on their analysis, this paper suggests the following ways to avoid some of the pitfalls of performance measurement and to create an enabling environment for primary care strengthening.

 First, accurate measurement for performance is needed. As Rasooly et al^7^ suggests, increasingly accurate and cost-effective use of more advanced test (such as hemoglobin A1C for diabetic patients) may be an important way to improve performance measurement.

 Second, information technology should facilitate verification of performance and identification of fraud. Rapidly developing in China are regional health information systems that cover nearly all of patients’ data related to health service utilization and health insurance claims. As a result, linking patients’ health records may make it convenient to tell a patient’s actual health and outcomes of NCDs management. Facial recognition has also been used for patients with hypertension and/or diabetes during follow-up visits provided by primary care providers. It helps make sure that local health workers actually perform follow-up visits and blood pressure measurement of and glycemia test.

 Third, for such measurement to be effective, it will likely require additional payment. Either increased input or reallocation of current fund will be needed for performance verification, information system, and using better indicators (including additional tests).

 Fourth, performance indicators for downstream outcomes may be used. For example, key indicators for primary care such as avoidable hospitalizations may need to be introduced at local and regional levels.

 Fifth, local stakeholders should be engaged in deciding both performance targets and their uses, so that performance measurement is realistic and meaningful. While new ways of measurement may squeeze out some frauds, they cannot eliminate room for gaming. Moreover, some of these technical solutions may not seem appropriate or feasible in certain areas. For local providers and bureaucrats to genuinely collaborate to improve primary care, they need to find such targets legitimate. Real challenges such as patients’ bypassing and lack of trust in primary care should be recognized as structural constraints for performance improvement. Using performance measurement along with efforts to empower and strengthen primary care service capacity may leverage the local potential to develop people-centered, high-quality care.

## Ethical issues

 Not applicable.

## Competing interests

 Author declares that he has no competing interests.

## Funding

 Jin Xu’s work is supported with the major research project “Building of and Database-construction for A Global Community of Health for All” (Grant No. 21ZDA130) sponsored by the National Social Science Fund of China. However, the funder played no role in the paper.
